# Genome-Wide DNA Methylation and Gene Expression Analyses of Monozygotic Twins Discordant for Intelligence Levels

**DOI:** 10.1371/journal.pone.0047081

**Published:** 2012-10-17

**Authors:** Chih-Chieh Yu, Mari Furukawa, Kazuhiro Kobayashi, Chizuru Shikishima, Pei-Chieng Cha, Jun Sese, Hiroko Sugawara, Kazuya Iwamoto, Tadafumi Kato, Juko Ando, Tatsushi Toda

**Affiliations:** 1 Division of Neurology/Molecular Brain Science, Kobe University Graduate School of Medicine, Kobe University, Kobe, Japan; 2 Keio Advance Research Centers, Keio University, Tokyo, Japan; 3 Department of Computer Science, Graduate School of Information Science and Engineering, Tokyo Institute of Technology, Tokyo, Japan; 4 Laboratory for Molecular Dynamics of Mental Disorders, RIKEN Brain Science Institute, Saitama, Japan; 5 Department of Molecular Psychiatry, Graduate School of Medicine, The University of Tokyo, Tokyo, Japan; 6 Faculty of Letters, Keio University, Tokyo, Japan; The George Washington University, United States of America

## Abstract

Human intelligence, as measured by intelligence quotient (IQ) tests, demonstrates one of the highest heritabilities among human quantitative traits. Nevertheless, studies to identify quantitative trait loci responsible for intelligence face challenges because of the small effect sizes of individual genes. Phenotypically discordant monozygotic (MZ) twins provide a feasible way to minimize the effects of irrelevant genetic and environmental factors, and should yield more interpretable results by finding epigenetic or gene expression differences between twins. Here we conducted array-based genome-wide DNA methylation and gene expression analyses using 17 pairs of healthy MZ twins discordant intelligently. *ARHGAP18*, related to Rho GTPase, was identified in pair-wise methylation status analysis and validated via direct bisulfite sequencing and quantitative RT-PCR. To perform expression profile analysis, gene set enrichment analysis (GSEA) between the groups of twins with higher IQ and their co-twins revealed up-regulated expression of several ribosome-related genes and DNA replication-related genes in the group with higher IQ. To focus more on individual pairs, we conducted pair-wise GSEA and leading edge analysis, which indicated up-regulated expression of several ion channel-related genes in twins with lower IQ. Our findings implied that these groups of genes may be related to IQ and should shed light on the mechanism underlying human intelligence.

## Introduction

Individual differences in cognitive abilities have long been an intriguing phenomenon to both lay people and scientists. Differences in intelligence, as measured by IQ tests, appear to remain relatively stable from childhood to late life [1], [2]. Further, the fact that intelligence, in the general population, has a normal distribution and long-term constancy allows for the assumption of the nature of IQ as being, at least partly, hereditary with quantitative trait features. In effect, the heritability of intelligence is estimated to be somewhere between 30% to over 80% in classical MZ twin versus dizygotic twin studies [3]–[5], marking it one of the highest among human quantitative traits.

Despite the significant role that genes are supposed to play in deciding an individual's cognitive abilities, progress to identify intelligence-related genes in healthy adults is not as promising [6], [7], and contrasts the increasing list of some 300 genes associated with mental retardation [8]. One possible explanation for the lack of replicated genetic findings in normal-range intelligence is the small effect size of each gene. The fact that genome-wide association studies in the scale of thousands of subjects identified no specific genetic variants associated with human intelligence implies that very large sample sizes are necessary to detect individual loci [9].

As underpowered studies face challenges in the attempt to identifying small effect quantitative trait loci, twin research might provide an alternative. Twin studies serve more than a means to estimate heritability of the aforementioned complex traits; they also present an important resource to evaluate quantitative trait loci. There are accumulating evidences that long thought to be genetically identical MZ twins manifest variations in copy number [10] or point mutations limited to one twin [11]; however, these genomic discordances have failed to explain all phenotypic discrepancies [12]. Differences in phenotypes between MZ twins can possibly be attributed to environmental factors, as well as epigenetic variants, which refer to the gene expression-related modifications that occur without altering DNA sequences. Epigenetic regulatory mechanisms have been reported to be associated with a number of biological phenotypes, including intelligence [13]. Among all known epigenetic mechanisms, DNA methylation has been the most extensively studied. Specifically, such studies have observed patterns of negative correlation between promoter region methylation and gene expression [14]. Additionally, studies based on phenotypically discordant MZ twins have revealed that those who are as best matched for genetics, gender, age, prenatal influences, and shared environment as nature could provide, are considered to possess the potential to detect epigenetic and transcriptomic differences, although research has yet to yield exclusive results [12], [15].

In the present study, we recruited 17 healthy MZ twin pairs who manifested discordance for intelligence between co-twins (i.e., more than 1 standard deviation (SD)). Regarding that MZ twins are identical in genetic composition, the IQ difference could be associated with environmental factors, via epigenetic mechanisms regulations. By analyzing array-based genome-wide DNA methylation and gene expression profiles, a novel list of genes with functions related to protein synthesis, DNA helicase activities, and ion channels was generated. To our knowledge, this is the first study tested for epigenetic and expression differences between phenotypically normal, yet discordant, MZ twins via genome-wide approaches.

## Results

### General characteristics of participants

This study is a part of the Keio Twin Study project [16]. We have collected 240 MZ twin pairs with IQ scores of both siblings tested (Fig.S1). Seventeen MZ twin pairs (5 male pairs and 12 female pairs) aged 25.1±2.5 years (range, 21–31 years), with IQ scores of normal range yet manifesting significant between co-twins differences formed the present study sample (Table 1). Mean IQ score of all 34 participants was 100.91±13.32 (range, 67–139), while the mean difference between co-twins was 20.76 points (range, 15–45). No documented psychological or physiological conditions were noted at the time of recruitment. Since there is no standard definition of discordance in MZ twins IQ scores, we adopted 15-point-difference as the principle inclusion criterion. Fifteen-point is not only one standard deviation of IQ scores in general populations, but it is also the average IQ difference for genetically unrelated individuals sharing family environments, whereas identical twins differ by only about 6 IQ points on average [17].

**Table 1:** Background data for participants.

			IQ Scores* (Points)		
Twin Pair ID	Age (year)	Gender	Twin A	Twin B	IQ score differences
1	31	F	99	82	17
2	30	F	110	91	19
3	22	M	82	112	−30
4	20	F	100	78	22
5	29	F	90	108	−18
6	20	F	86	104	−18
7	24	F	93	76	17
8	25	F	126	106	20
9	21	M	97	80	17
10	27	F	123	104	19
11	22	F	93	108	−15
12	23	M	97	117	−20
13	27	M	121	139	−18
14	23	M	126	108	18
15	26	F	112	67	45
16	26	F	108	90	18
17	24	F	110	88	22

* IQ scores calculated after participants took the full version of Kyodai Nx15- test.

### 27 genes identified by screening for epigenetically regulated candidate loci

We analyzed the methylation profiles of 25,500 human promoters utilizing methylated DNA enriched genomic DNA derived from peripheral blood cells. We used MAT (model-based analysis of tiling arrays) program [18] to identify genes that significantly differed between co-twins pairwisely. With a significance threshold set to *p*<10^−6^, a total of 27 genes were recognized in 13 of the 17 twin pairs; however, none was shared in plural pairs (Table S1). In addition, a moderate positive correlation of 0.417 (Spearman's rank correlation coefficient, *p* = 0.048) was found between the number of genes that epigenetically differed and pair-wise IQ differences (Fig. S2). On the other hand, after applying the log signal ratio (of the higher IQ twins to the lower IQ co-twins) to one-class *t*-test with the same significance threshold across all 17 twin pairs, we identified no locus manifesting significantly different methylation status.

### Validation of *ARHGAP18* by bisulfite sequencing and quantitative RT-PCR

**Figure 1:**
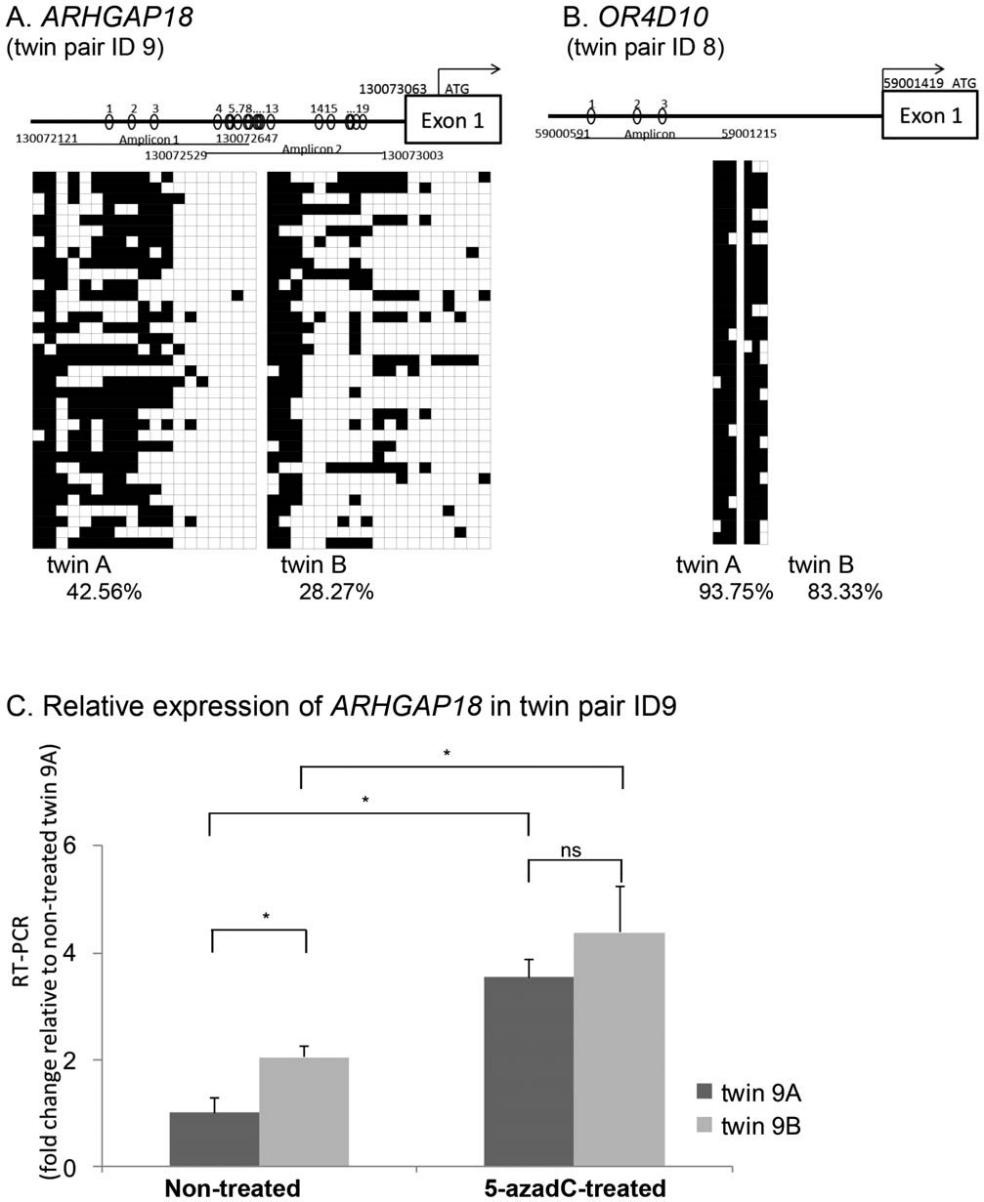
DNA methylation status of the 5′-regions of *ARHGAP18* and *OR4D10* analyzed by bisulfite sequencing along with the quantitative RT-PCR analysis for *ARHGAP18* expression. A, B. DNA methylation status analyzed by bisulfite sequencing. Schematic representation (top) for the relative position of CpGs within amplified regions and methylation profiling by bisulfite sequencing (bottom). The numbers at the ends of amplicons indicate the genome coordinates relative to the NCBI Build 36 genome assembly. Of all 27 loci identified by screening for epigenetically regulated candidate genes, A. *ARHGAP18* and B. *OR4D10* were validated by direct bisulfite sequencing. At least 30 clones were sequenced for each locus. Open squares indicate unmethylated CpG nucleotides and closed squares indicate methylated ones. Rows indicate the methylation status of each colony sequenced, while columns indicate the positions of CpG nucleotides.The percentages below refer to the ratio of CpG methylation. C. qRT-PCR analysis of *ARHGAP18* mRNA relative to *GAPDH* in the twin pair ID 9. *ARHGAP18* expression in twin 9A untreated with 5-azadC was normalized to 1. Error bars indicate ± SD. *P*-value was calculated using Mann-Whitney U test with asterisks indicating statistical significance (*p*<0.05). ns: not significant.

We performed a sodium bisulfite analysis to confirm the methylation status at the 27 gene loci putatively identified as being differentially methylated. After sequencing at least 30 clones for each locus, statistically significant difference in methylation status between co-twins was validated on two genes, *ARHGAP18* (Rho GTPase activating protein 18, *p* = 5.12806×10^−8^; chi-square test) and *OR4D10* (olfactory receptor 4D10, *p* = 0.0234658; chi-square test) (Fig. 1A and B).

After confirmation of methylation status, we correlated the data with their expression levels using total RNA derived from lymphoblast cell lines by qRT-PCR. The observed reduction in DNA methylation status of *ARHGAP18* was correlated with its increased expression level in the subject with lower IQ scores of the twin pair from which the gene was identified (2.04 fold, *p* = 0.024767; Mann-Whitney U test) (Fig. 1C, left graph). Increased transcription levels were observed after a 3-day treatment of the demethylating agent 5-aza-2-deoxycytidine (5-azadC), and the difference between the twins was eliminated, suggesting that the expression is regulated by the methylation of the promoter (Fig. 1C, right graph). Meanwhile, no expression of *OR4D10* could be detected in the lymphoblast cell lines.

### No gene manifesting statistically significant differences between the group of twins with higher IQ and that of their co-twins after the expression array analysis

Apart from the epigenetic approach described above, we used an expression microarray analysis to directly compare the genome-wide gene expression profiles of the higher IQ twins versus their lower IQ co-twins. At first, principal component analysis (PCA) was performed to facilitate visualization of the relationships between groups (Fig. S3). As a result, the two groups compositing twins with higher or lower IQ scores could not be readily distinguished. Likewise, the dendrograms, produced by hierarchical clustering, also failed to demonstrate differences in general expression patterns between these two phenotypic groups (Fig. S4). Next, ANOVA analysis was applied to identify whether there were differentially expressed genes between these two groups; however, no gene met the criteria of a FDR (false discovery rate)-adjusted *p* of 0.05. A one-class *t*-test analysis with multiple sample correction was conducted across all log ratios. Similarly, no significantly differentially expressed gene was identified.

### Candidate genes list resulted from direct pair-wise comparison of the expression array data

Next, we applied a direct pair-wise comparison to focus on the genes up-regulated in the same tendency. Fold-change values of the expression levels of all genes were first calculated for each twin pair, from which genes with a fold-change value more than 2 were included (Dataset S1). We then generated a list of candidate genes by picking up those replicated in most twin pairs (Table 2). *UCHL1* (ubiquitin carboxyl-terminal esterase L1), along with the other 7 genes, were found to have higher expression levels in the higher IQ twins of at least 4 pairs, while 6 genes were up-regulated in some lower IQ twins.

**Table 2:** List of genes having the same tendency of expression level in most twin pairs.

Up-regulated in co-twins with	Gene	Shared by twin pair ID
Higher IQ scores	*GTSF1*	1,2,7,11,14,15,16
	*AK3L1*	1,3,9,11,17
	*PRKCH*	2,9,11,14,17
	*CDRT1*	2,9,10,11,14
	*LRIG3*	1,2,9,15
	*VSIG6*	2,10,11,13
	*SNORA20*	3,6,10,12
	*UCHL1*	8,9,11,15
Lower IQ scores	*CD96*	1,2,4,9,14
	*CXCL10*	1,6,9,11
	*CDC42BPA*	1,2,6,9
	*CXCR4*	1,2,6,14
	*EPS8*	2,9,10,16
	*FAM169A*	3,9,11,15

### Identification of 3 genes with borderline significance by grouping samples according to individual gene expression level

**Figure 2:**
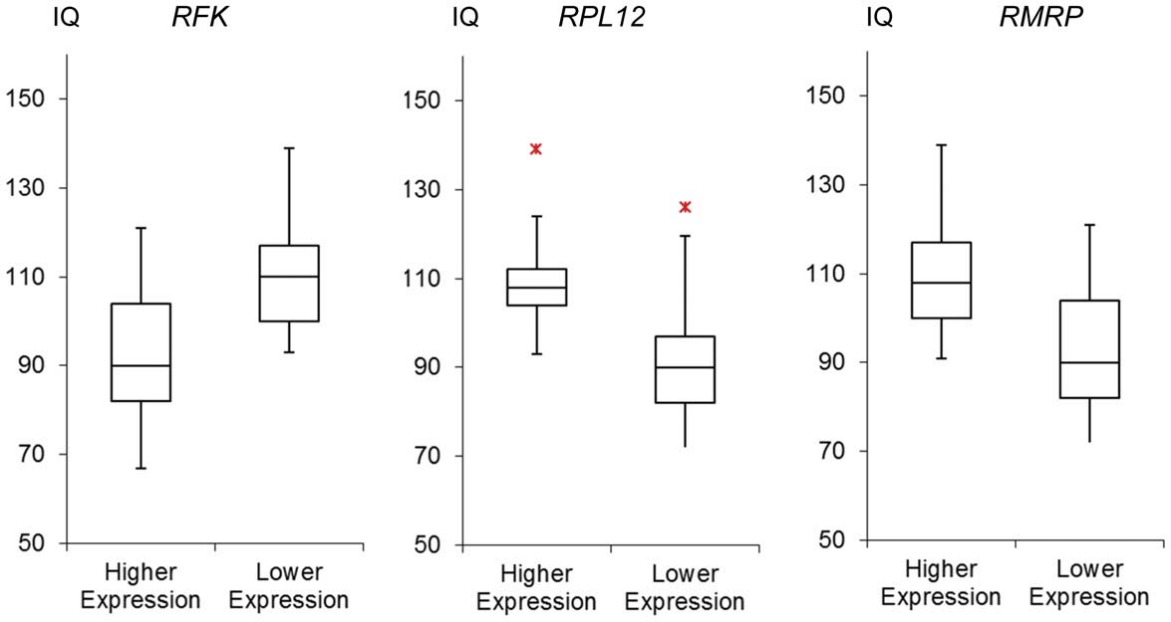
Three genes manifesting borderline significance by grouping samples according to individual gene expression level. IQ scores in subjects were grouped according to the relatively higher (Higher Expression) or lower (Lower Expression) expression levels within each twin pair. Three genes, *RFK*, *RPL12*, and *RMRP*, manifested the smallest *p* yet failed to meet the cutoff value of p = 10^−6^. Data are presented as box plots (minimum, 25% quartile, median, 75% quartile, maximum). The red asterisks indicate maximum outliners.

To further increase the possibility of identifying candidate genes, we performed an analysis based on individual gene expression level. After dividing the twin siblings of each pair into higher and lower expression groups according to the expression level of every gene, a paired t-test was carried out to determine if there was a significant difference between the mean IQ scores of the two groups. Whereas not a single gene reached the corrected cutoff *p* of 10^−6^, 3 genes, *RFK* (riboflavin kinase), *RPL12* (ribosomal protein L12), and *RMRP* (RNA component of mitochondrial RNA processing endoribonuclease), manifested borderline significance (Fig. 2). The twins manifesting up-regulated expression level of *RFK* showed the tendency to have lower IQ scores than their co-twins, while *RPL12* and *RMRP* might likely to contribute to higher intelligence.

### Identification of 4 differentially expressed gene sets by GSEA

**Figure 3:**
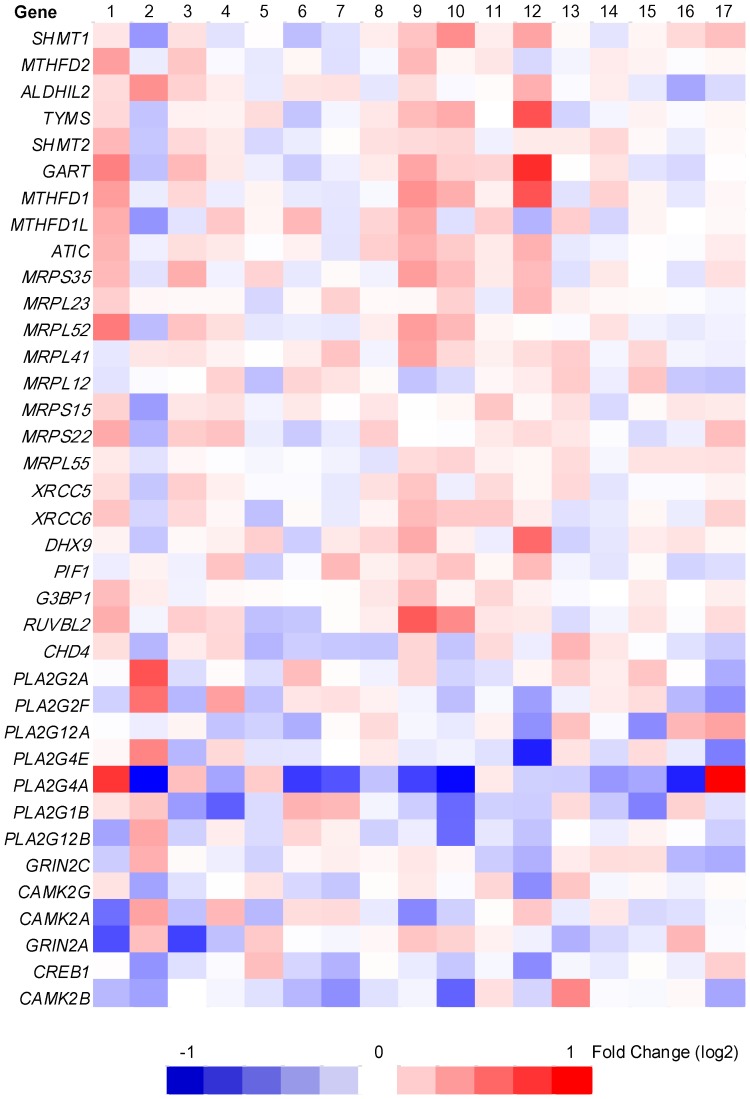
Heat map of the 37 genes with core enrichment from up-regulated genes sets. GSEA analysis was carried out to identify if any pre-defined gene set showing different expression levels between the group of higher IQ twins and the group of their lower IQ co-twins. Gene set databases BioCarta, KEGG, Reactome, and Gene Ontology were applied separative. Gene sets meeting the cutoff FDR *q-*value of 0.25 were subjected to leading edge analysis to determine the genes with core enrichment. The result was a list of 37 genes and we generated a heat map accordingly. Red and blue cells signify genes that were either up- or down-regulated, respectively, after the expression levels of the twins with higher IQ scores compared to their co-twins. The scale represents fold changes in log2 values, according to the color map at the bottom of the figure. The general tendency of higher expression levels in twins with higher IQ scores from the gene *SHMT1* to *CHD4*, and their lower expression levels from the gene *PLA2G2A* to *CAMK2B*, was visualized.

It remained possible that functionally-related genes might have important gene expression changes in a set-wise matter without any individual transcript meeting the criteria of significance. Using GSEA [19] under the cutoff FDR *q*-value of <0.25, we denoted 1 and 4 up-regulated gene sets from KEGG and Gene Ontology (GO) database respectively in the group of higher IQ twins, while 1 gene set each from KEGG and Reactome pathway database was found to be up-regulated in the group of lower IQ twins (Table S2 and Fig. 3). From the results employing GO database, the leading edge analysis revealed 8 genes (*MRPS35*, *MRPL23*, *MRPL52*, *MRPL41*, *MRPL12*, *MRPS15*, *MRPS22*, and *MRPL55*) with core enrichment in the gene sets “Organellar Ribosome,” “Ribosomal Subunit,” and “Mitochondrial Ribosome”, whereas the fourth gene set “ATP-dependent DNA Helicase Activity” was comprised of 7 other genes (*XRCC5*, *XRCC6*, *DHX9*, *PIF1*, *G3BP1*, *RUVBL2*, and *CHD4*) with core enrichment. By utilizing Reactome database, all 6 genes from the gene set “Reactome CREB phosphorylation through the activation of CAMKII” were enriched.

A different approach was carried out to the same end. Specifically, we performed a GSEA on each twin pair. Depending on the twin pairs analyzed and pathway databases used, as many as 284 gene sets were found to be significantly different between the siblings (Dataset S2, S3, S4, S5). Gene sets replicated in most twin pairs were listed. In general, pathways related to DNA replication, ribosomes, and proteases were found in higher IQ twins of most twin pairs, while cell signaling associated ones tended to be up-regulated in lower IQ twins (Table 3). In order to focus on those genes which effectively contributed to the enrichment of each given gene set, we first generated up- and down-regulated leading edge subsets for each twin pair, and then extracted those genes that were nominated most often across plural twin pairs (Table 4). Up-regulation of *IGF1* was found in the higher IQ twins of 4 pairs, whereas potassium channel-coding genes *KCNE2* and *KCNQ3*, along with an acetylcholine receptor-coding gene *CHRNA2* manifested higher expression levels in some lower IQ twins. Among all the candidates, *IGF1* was selected for further analysis for its important role in growth and development [20]. We performed bisulfite sequencing of the promoter regions for the 4 twin pairs manifesting an up-regulated expression level in the higher IQ siblings. However, no significant differences were noted in the methylation status of the 2 promoter domains (P1 and P2) between the siblings (Table S3).

**Table 3:** Summary of the most commonly shared gene sets showing the same tendency (up-regulated in the higher IQ twin or in the lower IQ twin) according to the pair-wise GSEA test.

Up-regulated in	Gene sets database	Gene set	Shared by twin pair ID
Higher IQ twins	BioCarta	MCM pathway	1,9,12,17
	KEGG	Proteasome	1,3,9,10,17
		Oxidative phosphorylation	1,9,10,12,16
		Ribosome	1,6,9,10,12,16
		DNA replication	1,3,8,9,10,12,17
		Valine leucine and isoleucine degradation	1,3,10,12,17
		Mismatch repair	1,3,9,12,17
		Peroxisome	1,9,10,12,17
	Reactome	RNA polymerase I promoter opening	1,3,9,11,12,13,16,17
		Electrotransport chain	1,3,9,10,12,13,16,17
		Packaging telomere ends	1,3,9,11,12,16,17
		Telomere maintenance	1,3,9,11,12,16,17
	Gene Ontology	Structural constituent of ribosome	9,10,12,17
		Microbody	1,9,12,17
		Peroxisome	1,9,12,17
		S-phase of mitotic cell cycle	1,8,15,17
		Small conjugating protein specific protease activity	1,9,15,17
Lower IQ twins	BioCarta	n/a	n/a
	KEGG	Neuroactive ligand receptor interaction	1,3,16,17
		Taste transduction	1,3,9,13,17
	Reactome	Cell-cell adhesion systems	1,3,9,17
	Gene Ontology	Anion transport	1,5,16,17
		Cation channel activity	1,9,16,17
		Cell-cell signaling	1,9,16,17
		Collagen	1,9,16,17
		Extracellular matrix part	1,9,16,17
		Extracellular region part	1,9,16,17
		G-protein coupled receptor protein signaling pathway	1,9,16,17
		Gated channel activity	1,9,16,17
		Intercellular junction	6,9,16,17
		Metal ion transmembrane transporter activity	1,9,16,17
		Second messenger mediated signal	1,9,16,17

n/a indicates no gene set was shared by at least 4 twin pairs.

**Table 4:** Up-regulated genes with core enrichment shared by co-twins of multiple pairs.

Up-regulated in	Gene Sets database	Gene	Shared by twin pair ID
Higher IQ twins	BioCarta	*CASP6*	1,10,12,17
		*CCNE1*	1,9,12,17
		*IGF1*	1,2,7,17
		*IL1a*	2,7,8,15
		*LTA*	2,8,15,17
		*C7*	2,3,8,15
		*IL6*	2,3,7,15
		*STAT4*	3,8,10,17
	KEGG	*MOM6*	1,3,8,9,12,17
		*RPA2*	1,3,8,9,12,17
		*POLA2*	1,3,8,9,12
		*PRIM2*	1,3,8,9,12
		*MOM2*	1,3,8,9,12
		*LTA*	2,3,10,15,16
		*SDHB*	1,3,9,10,16
		*POLE2*	1,3,8,9,17
		*COX7C*	1,9,10,12,16
		*COX8A*	1,9,10,12,16
		*NDUFB7*	1,9,10,12,16
		*NDUFA8*	1,9,10,12,16
		*NDUFA7*	1,9,10,12,16
		*SSBP1*	1,8,9,12,17
	Reactome	*NUP93*	1,3,9,10,11,12,17
		*POLE2*	1,3,9,11,16,17
		*POLR2L*	1,3,9,11,12,16
		*POLR2G*	1,3,9,12,16,17
		*PSMD14*	1,3,9,11,12,17
		*PSMC6*	1,3,9,11,12,17
		*RPA2*	1,3,9,11,12,17
		*NUP85*	1.3.9.10.11.12
		*NUP37*	1.3.9.11.12.17
		*NUP205*	1.3.9.11.12.17
		*CDC6*	1.3.9.11.12.17
		*CDC25A*	1.3.9.11.12.17
		*ORC4L*	1.3.9.11.12.17
		*KMCM6*	1.3.9.11.12.17
		*PRIM1*	1.3.9,11,16,17
	Gene Ontology	n/a	n/a
Lower IQ Twins	BioCarta	n/a	n/a
	KEGG	*EP300*	1,3,6,12,13
		*ACTN1*	1,6,12,13,14
		*PLOB2*	1,3,8,13
		*PIK30G*	1,6,8,13
		*CREBBP*	1,3,12,13
		*MAPK9*	1,2,6,13
		*LEF1*	1,3,6,12
		*PIK3R3*	2,6,8,13
		*AKR104*	3,8,16,17
		*PARD3*	6,8,12,16
	Reactome	*MNAT1*	2,6,7,14
		*RFC2*	2,6,7,14
		*PRIM1*	2,6,7,14
		*PRIM2*	2,6,7,14
	Gene Ontology	*CHRNA2*	1,9,16,17
		*KCNE2*	1,9,16,17
		*KCNQ3*	1,9,16,17
		*INHBA*	1,9,14,17
		*SLC34A3*	1,5,16,17

n/a, no gene was shared by at least 4 twin pairs.

## Discussion

Given that the concept of general cognitive ability, designated as *g*, has been widely accepted to depict a near-universal positive covariation among diverse measures of cognitive abilities, naming even one genetic locus that is reliably related with normal-range intelligence remains challenging [21]. IQ is easy to quantify and compare among different individuals. Although not conclusively, the substantial *g*-loading for IQ [22] justifies its role to represent the general intelligence levels. Benefiting from the extraordinary similarities in genomic constitution and environmental factors, studies based on discordant monozygotic twins, even with limited sample size (as small as 20–50 twin pairs), are capable of uncovering phenotype-associated epigenetic changes independent of underlying sequence variance [23]. By successfully recruiting 17 pairs of identical twins discordant for intelligence levels, we shall have a modest power in the identification of intelligence-related epigenetic differences. To our knowledge, this is the first genome-wide methylation and gene expression study administering the characteristics of monozygosity to access the epigenetic and expression changes for a quantitative trait.

Researchers have reported that patterns of epigenetic modifications in MZ twins diverge as they age [24]. Provided that all 17 twin pairs in this study were in early adulthood, it might not be surprising that only few loci revealed significant differences in methylation status. Of the 27 candidate loci nominated by promoter-arrays-based methylation analyses, bisulfite sequencing successfully validated only 2 genes. One explanation of the discrepancy between these two methods is that we adopted a less stringent criterion of *p*-value (10^−6^, instead of a Boferroni corrected p-value of 10^−8^ considering that GeneChip Human Promoter 1.0 Array contains 4.6 million probes). As a result, we were able to detect more candidate loci from the practically congruent MZ twin samples, only at the expense of precision rate. The technical limitations of MethylMiner collection might also contribute to false positives. After bisulfite sequencing and qRT-PCR validation, we identified one candidate gene, *ARHGAP18*, which encodes one of the Rho GTPase-activating proteins (GAPs) that modulate cell proliferation, migration, intercellular adhesion, cytokinesis, proliferation differentiation, and apoptosis [25]. Mutations in a handful of Rho-linked genes were documented to be associated with X-linked mental retardation [8], by which the importance of GAP activity in normal neuronal functions was proposed [26]. Notwithstanding the identification of *ARHGAP18* in a genome-wide association study for schizophrenia [27], it had not been previously connected to cognitive abilities until the present study.

We were not able to identify a single gene that displayed significantly different expression level between the group of twins with higher IQ scores and their co-twins. From the list of candidate genes generated by direct pair-wise comparison, UCHL1 is a brain-specific de-ubiquitinating enzyme. While the substrates are still unknown, loss of its enzyme activity has been reported in neurological diseases such as Alzheimer's disease and Parkinson's disease [28]. In a different approach, *RFK*, *RPL12*, and *RMRP* showed borderline significance. *RFK* encodes riboflavin kinase, an essential enzyme to form flavin mononucleotide, is important in a wide range of biological metabolisms [29]. *RPL12* encodes a ribosomal protein of the 60S subunit, while *RMRP* encodes the RNA component of mitochondrial RNA processing endoribonuclease. Although none of these 3 genes had ever been connected with cognitive functions, it remains possible that their biophysical characteristics might become more pronounced in cells having as high a metabolic rate as neurons.

In the gene set based approach, GSEA of between-group and between co-twin comparisons revealed several mitochondrial ribosomal protein-coding genes. Mitochondria, which are responsible for most of the energy requirement for cellular metabolism, have their own translation system for the 13 proteins essential for oxidative phosphorylation in mammals. All 78 human mitochondrial ribosome proteins are translation products of nuclear genes, of which some were identified as candidate genes for several congenital diseases [30]. No exclusive conclusion about the connection of mitochondrial ribosomal function and cognitive ability could be drawn before being further validated. Nevertheless, we hypothesized that, for the highly differentiated and high energy-demanding central nervous system, essential proteins for mitoribosome function might play a role in the maintenance of neuronal biological processes.

Apart from mitochondrial ribosomal protein-related gene sets, “ATP-dependent DNA Helicase Activity” from the GO database was also found. DNA helicases are molecular motor proteins that use nucleoside 5′-triphosphate hydrolysis as a source of energy to open energetically stable duplex DNA into single strands. As such, they are essential in almost all aspects of cellular DNA machinery including DNA replication, repair, recombination, and transcription [31]. Of which, *XRCC5* and *XRCC6* encode the two subunits of the Ku protein, which plays an important role in the repair of double-stranded DNA breaks and telomere protection [32]. In neurodegenerative diseases, such as Alzheimer's disease, where cellular damage due to oxidative stress is proposed to contribute to pathophysiology, reduced Ku protein expression and its DNA binding activity have been thought to be involved [33]. Of the remaining five helicases denoted, *G3BP1* was demonstrated to play an essential part in proper embryonic growth and neonatal survival [34]. Although the direct associations between these seven genes and cognitive abilities have not been depicted, mutations in a list of DNA repair-related genes have already been reported to cause mental retardation [8]. As such, one hypothesis we proposed here is that the up-regulated expression of these helicases might provide better protection from oxidative damages and, thus, improve neuronal function and survival, which could bring forth higher levels of intelligence (or in other words, less compromised) as a phenotype.

On the other hand, the pair-wise GSEA, along with leading edge analysis, identified *CHRNA2* which encodes the α2 subunit of nicotinic acetylcholine receptors (nAChRs). Initially related to nicotine dependence, the role of nAChRs in cognitive performance has gained attention because nicotine is considered a powerful enhancer of cognitive capabilities [35] via the interaction of nicotine and nAChRs [36].

Additionally, two potassium voltage-gated channel-coding genes, *KCNE2* and *KCNQ3*, were identified. Voltage-gated ion channels possess diverse functions, include regulating neurotransmitter release, heart rate, insulin secretion, neuronal excitability, epithelial electrolyte transport, and smooth muscle contraction. By assembling with KCNQ2 or KCNQ5, KCNQ3 forms the M channel, a slow activating and deactivating potassium channel that plays a critical role in the regulation of neuronal excitability [37]. In addition to being identified as one cause for a dominantly inherited form of human generalized epilepsy, called benign familial neonatal convulsions, the electrogenic characteristics of KCNQ/M channels have importance in controlling intrinsic firing patterns of principal hippocampal neurons, thus, further modulating hippocampal learning and memory [38]. Of note, rats treated with Linopirdine, an M channel-specific inhibitor, demonstrated improved performance in various tests of learning and memory [39].

Identified by utilizing BioCarta pathway database, *IGF1* manifested up-regulation in higher IQ twins. The insulin-like growth factor (IGF) system is important in growth and development. While the exact mechanism remains unknown, the growth hormone (GH) and IGF-1 axis has been reported to play a role in the reduction of cognitive functions in aging population and patients with GH deficiency [20]. Methylation status of its promoter regions was studied, yet no difference was discerned between the twins. It is possible that *IGF1* is under some other epigenetic regulation, considering the actual mechanisms responsible for the cell type-specific expression patterns of this gene remain to be elucidated [40].

Since intelligence is a complex trait associated with many genes of small effect [41], [42], it was not surprising that we failed to identify a single gene manifesting prevailing expression changes across all 17 twin pairs. We also noticed that by microarrays none of the candidate genes identified by methylation analyses was listed in the results of expression studies. A reason of the discrepancy of these two methods may be the lack of comprehensive accession to all known epigenetic regulations. DNA methylation at CpG sites across promoter regions has been deeply studied, while a number of other epigenetic regulatory mechanisms are also found to modulate gene expression [22]. Furthermore, ever since the genome-wide, single-base human DNA methylome mapping became possible, the correlation of methylation status of gene bodies and expression levels has been gaining attention [43]. It has been documented that DNA methylation of gene bodies is associated with gene activity. Therefore, it is possible that the intelligence-related expression profiles were subjected to this novel epigenetic regulation.

The present study has its conceptual and technical limitations. Conceptually, we hypothesized that changes in methylation status and expression levels could be captured in genomic DNA and total RNA extracted from whole blood and derivative lymphoblastic cell lines, respectively. Given the inability to probe DNA methylation status or gene expression in the human brain, except in postmortem studies, human blood is commonly used in transcriptional studies of various diseases including psychiatric disorders [44]. Although there is still no consensus regarding blood-based gene expression profiles as good surrogates for addressing neuroscientific research, the moderate correlation between transcripts in whole blood and the central nervous system makes it an accessible alternative [45], [46]. Several studies utilizing similar strategies to study twin pairs discordant for psychiatric disorders through comparisons of CpG islands methylation of peripheral blood cells or lymphoblast cell lines had detected a number of disease-associated epigenetic changes [47]–[49]. Moreover, it has been documented that the methylation changes of large-scale domains are linked to cell-specific differentiation [50]. Several functional gene sets we found (i.e., “cation channel activity”, “cell-cell signaling”, “extracellular region part”, “G-protein coupled receptor protein signaling pathway”, and “gated channel activity”) were observed within neuronal highly methylated domains. The association between expression difference observed in lymphoblast cell lines and neuron-specific methylation patterns implies that these candidate gene sets are more likely to reflect the true differences in brains. Technical limitations of this study include low fold-change differences in expression levels manifested between co-twins. We did not carry out qRT-PCR for the genes identified by GSEA, considering that differences of less than 1.5-fold are thought to be beyond the limit of reproducibility [51].

Reverse causality should be considered in epigenetic studies, considering all known epigenetic marks are influenced by environmental exposures including diet, smoking, alcohol consumption, stress, or physical activities [52], It might be plausible that the changes we observed in this study resulted from the divergent lifestyle choices by subjects with different levels of intelligence, and not that these epigenetic changes caused the twins to differ.

Here, we presented the first study that used genome-wide epigenetic and transcriptomic profiling to identify epigenetic changes related to the discordance between MZ twins with normal-range intelligence. A list of new candidate genes possibly related to cognitive abilities was generated while further replications and functional analysis remain necessary.

## Materials and Methods

### Ethics statement

This study was conducted according to the principles expressed in the Declaration of Helsinki. Attendance was voluntary, and signed informed consent including information on genetic analyses was obtained from all participants. The Ethical Committees of Kobe University Graduate School of Medicine and Keio University Faculty of Letters approved study protocols.

### Samples

A sub-sample of 326 twin pairs of twins from the Keio Twin Project were invited to Keio University, where the Kyodai Nx15-, one of the most often used group intelligence tests in Japan, was applied. The zygosity of participants was diagnosed by 15 polymorphic STR loci (AmpF*ℓ*STR identifier kit, Applied Biosystems). Among the 240 pairs to have monozygosity, 34 MZ twin pairs who manifested differences in IQ score of more than 15 points between co-twins. One pair was excluded for a lower-than-normal IQ score (52 points). From the17 of the remaining 33 twin pairs, who agreed to participate in the study, peripheral blood was drawn and B-lymphoblastoid cell lines were established. For the treatment of 5-azadC (WAKO), a daily aliquot of 5 mM stock solution was added to flasks and thoroughly resuspended (final concentration of 1 µM). Cells were harvested after 3 days from the start of treatment.

### Nucleic acids extraction

For human promoter microarrays and bisulfite genomic sequencing, genomic DNA was extracted from the blood via established methods. For gene expression microarrays and quantitative RT-PCR, total RNA was isolated using RNeasy Plus Mini kit (QIAGEN) from B-lymphoblastoid cell lines.

### DNA methylation profiling

One microgram of genomic DNA was sonicated and subjected to methylated DNA enrichment using the MethylMiner methylated DNA enrichment kit (Invitrogen) as per the manufacturer's instructions. The methylated DNA fragments, amplified by the GenomePlex WGA reamplification kit 3 (SIGMA) and supplemented with dUTP, were further purified using the QIAquick PCR purification kit (QIAGEN).

According to Affymetrix's chromatin immunoprecipitation assay protocol, enriched methylated DNA was hybridized to GeneChip Human Promoter 1.0R arrays (Affymetrix), which comprised a coverage of over 25,500 human promoter regions.

AGCC (Affymetrix GeneChip Command Console)-format CEL files were first created, and then converted to GCOS (GeneChip Operating Software, Affymetrix)-format CEL files. For the pairwise analyses, paired CEL files were imported into MAT software to specify candidate regions (approximately 600 base pairs in length) with significantly different probe intensities between co-twins (*p*<10^−6^).

To detect candidate loci across all 17 twin pairs, we utilized Partek Genomic Suite 6.5 software (Partek) to import the CEL files, and have the data converted to log_2_ values after normalized by the RMA (Robust Multichip Averaging) algorithm. After the signal from each probe for the higher-IQ sibling was subtracted from that of the lower-IQ co-twin across all probes, one-class *t*-test with statistical parameters set at *p*<10^−6^ was carried out to detect significant regions.

### Bisulfite sequencing

Genomic DNA was bisulfite-treated using the Methylcode bisulfite conversion kit (Invitrogen) as per the manufacturer's instructions. Amplification was performed with Takara LA Taq polymerase, with converted DNA-specific primers that were designed using MethPrimer. The amplicons were cloned into vectors using a TOPO TA cloning kit (Invitrogen). We performed direct sequencing of the plasmid DNA that was isolated using PI-200 auto-plasmid-isolator (KURABO) via the ABI 3730xl sequencing system (Applied Biosystems).

### Quantitative RT-PCR

Two micrograms of the total RNA was subjected to reverse transcription using the SuperScript III first-strand synthesis system for RT-PCR (Invitrogen). Gene target amplifications, using Takara SYBR premix Ex Taq, were performed in triplicate in a matter of a serial 10 fold dilution. Housekeeping gene *GAPDH* served as the internal control gene. Mann-Whitney U test was performed to compare the relative expression levels between co-twins.

### Gene expression profiling

We processed 300 nanograms of total RNA using the Ambion WT expression kit and the Affymetrix GeneChip WT terminal labeling kit according to the manufacturers' recommended methods. Hybridization and scanning of GeneChip Human Gene 1.0 ST arrays (Affymetrix), which comprised of more than 28,000 gene-level probe sets, were performed as per the manufacturer's instructions. Partek Genomic Suite 6.5 software was used to import AGCC-format CEL files and normalize the data according to the RMA algorithm. ANOVA with an FDR-adjusted *p* set to 0.05 was used to determine those probe sets that were significantly different between the groups of twins with a higher IQ and their lower IQ co-twins. A one-class *t*-test analysis with multiple sample correction was conducted across all log_2_ ratios (higher-IQ twin/lower-IQ co-twin) for all 17 twin pairs. We also carried out pair-wise comparison for the expression array data and then included genes with a fold-change value more than 2. Genes replicated in the same tendency (up-regulated in the higher IQ twins, or up-regulated in the lower IQ twins) in most pairs were listed.

In another approach, the twins of each pair were categorized into higher and lower expression groups according to the expression level of every individual gene. A paired t-test was carried out to compare the mean IQ scores of the two groups. Corrected *p* of 10^−6^ was applied as the cutoff to define being positive.

GSEA was performed for functionally related genes across a spectrum of gene sets of C2 curated gene sets including BioCarta gene sets (217 gene sets), KEGG gene sets (186 gene sets), and Reactome gene sets (430 gene sets), and C5 GO gene sets (1,454 gene sets) separately. Pre-ranked gene lists, including lists with up-regulated/down-regulated genes in the group of twins with higher IQ scores sorted according to the *p* calculated by a between-group ANOVA test, and lists for each twin pair with genes sorted by between-sibling fold-change values, were constructed for the analyses. Gene sets with FDR *q*-value<0.25 after 1,000 permutation cycles were considered significantly enriched. Lists of leading edge subset genes, the cores of gene sets that account for the enrichment signal, were then generated. To create the list of enriched genes shared by plural twin pairs, the upper 100 enriched genes of each pair were included in the test.

## Supporting Information

Dataset S1 **Pair-wise comparison of expression array data.** Genes with a fold-change value >2 were included. The positive value of fold-change designates up-regulation in the higher IQ twin, while the negative value designates the other way round. n/a indicates no gene matched the fold-change cutoff value.
(XLS)

Dataset S2 **Pair-wise GSEA results using BioCarta database.** Gene sets with a FDR *q*-value<0.25 were included. n/a indicates no gene set matched the FDR cutoff value.
(XLS)

Dataset S3 **Pair-wise GSEA results using KEGG pathway database.** Gene sets with a FDR *q*-value<0.25 were included. n/a indicates no gene set matched the FDR cutoff value.
(XLS)

Dataset S4 **Pair-wise GSEA results using Reactome database.** Gene sets with a FDR *q*-value<0.25 were included. n/a indicates no gene set matched the FDR cutoff value.
(XLS)

Dataset S5 **Pair-wise GSEA results using GO database.** Gene sets with a FDR *q*-value<0.25 were included. n/a indicates no gene set matched the FDR cutoff value.
(XLS)

Figure S1 **Scatterplot of the 240 MZ twin pairs IQ scores.** This diagram provides an overview of the IQ distribution for all 240 MZ twins from the Keio Twin Study. Each circle identifies one twin pair with its x-coordinate and y-coordinate representing, respectively, the IQ score of Twin A and Twin B. With the black line standing for regression, the correlation coefficient of 0.72 suggests the similarities between twins. Circles located outside of the space between two blue lines indicate twin pairs manifesting between-sibling IQ differences larger than 15 points and were considered to be recruited, while the red arrowhead points to one pair being excluded as a possible subject for a lower-than-normal IQ score.
(TIF)

Figure S2 **Positive correlation between IQ scores differences and the number of loci different in methylation status.** The positive correlation between the number of loci with significant differences in methylation patterns and the differences of IQ scores of the twin pairs was visualized. Twin pairs with larger differences in IQ scores tended to have more loci identified by screening for epigenetically regulated genes.
(TIF)

Figure S3 **PCA results for the expression profiles of 17 twin pairs.** Principle components ranked from the highest variance are named accordingly as PC 1^st^ and PC 2^nd^. The PCA projection maps these two components data to 2 dimensions for visualization. In the scatter plots, each point represents a sample. The color of the symbol represents the relative IQ scores with red as higher and blue as lower. The number on each symbol indicates the twin pair ID. In contrast to the similarity between co-twins from each pair, no apparent gathering pattern could be recognized by the relative IQ scores.
(TIF)

Figure S4 **Clustering analysis for the expression profiles of 17 twin pairs.** Clustering analysis for the list of 644 genes manifesting more than a 1.1-fold change between the groups of twins with higher IQ scores and the groups of their co-twins under ANOVA analysis was performed. The number below each symbol indicates the twin pair ID. The color red and blue of the symbol indicate respectively the higher IQ and the lower IQ twin of each twin pair. The scale represents fold changes, according to the color map at the bottom of the figure. No apparent clustering could be recognized.
(TIF)

Table S1 **Summary of candidate loci with methylation changes identified by promoter DNA methylation patterns and their bisulfite sequencing result.**
(DOC)

Table S2 **Up-regulated gene sets in the group of twins identified by GSEA (FDR** ***q*** **-value<0.25).**
(DOC)

Table S3 **Methylation profiling by bisulfite sequencing for** ***IGF1*** **(Chr2:101335584–131398508) in Twin Pair ID 7.**
(DOC)
