# Supplementary figures and images for: Genome-Wide DNA Methylation and Gene Expression Analyses of Monozygotic Twins Discordant for Intelligence Levels
Source: PLoS One. 2012 Oct 17;7(10):e47081. doi: 10.1371/journal.pone.0047081 (PMC3474830; doi:10.1371/journal.pone.0047081)

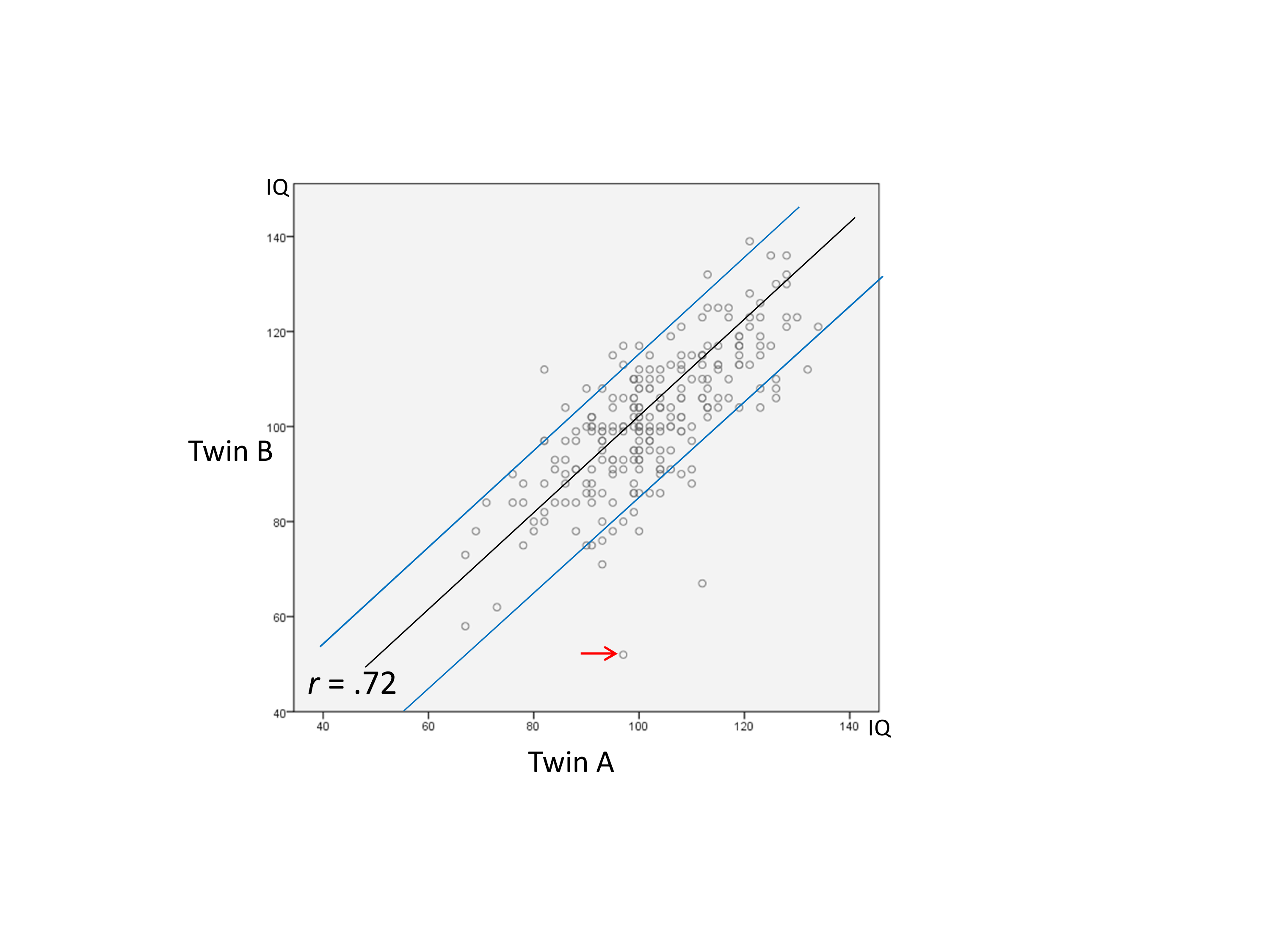

Supplement: Figure S1 — Scatterplot of the 240 MZ twin pairs IQ scores. This diagram provides an overview of the IQ distribution for all 240 MZ twins from the Keio Twin Study. Each circle identifies one twin pair with its x-coordinate and y-coordinate representing, respectively, the IQ score of Twin A and Twin B. With the black line standing for regression, the correlation coefficient of 0.72 suggests the similarities between twins. Circles located outside of the space between two blue lines indicate twin pairs manifesting between-sibling IQ differences larger than 15 points and were considered to be recruited, while the red arrowhead points to one pair being excluded as a possible subject for a lower-than-normal IQ score. (TIF) [file pone.0047081.s006.tif]

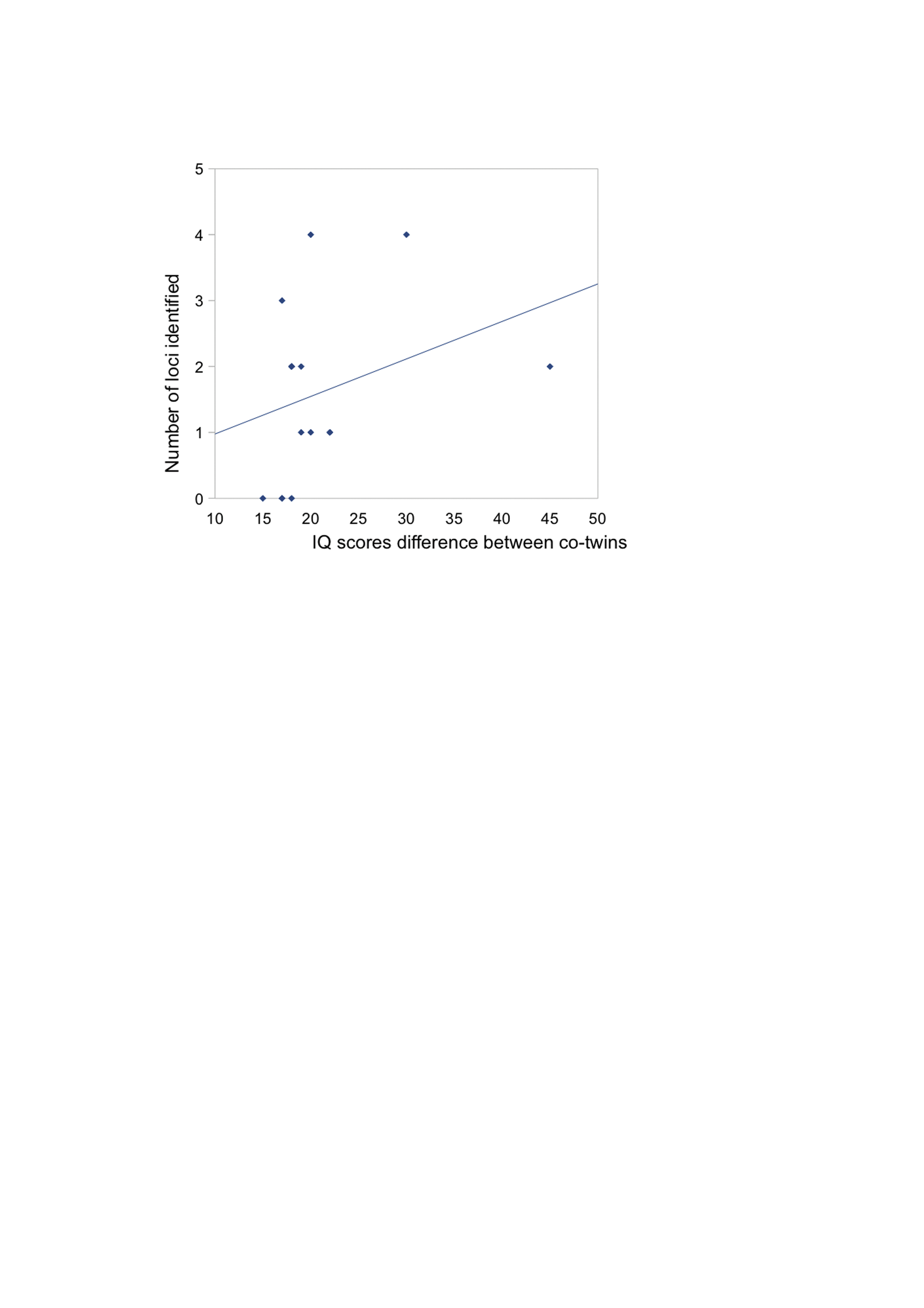

Supplement: Figure S2 — Positive correlation between IQ scores differences and the number of loci different in methylation status. The positive correlation between the number of loci with significant differences in methylation patterns and the differences of IQ scores of the twin pairs was visualized. Twin pairs with larger differences in IQ scores tended to have more loci identified by screening for epigenetically regulated genes. (TIF) [file pone.0047081.s007.tif]

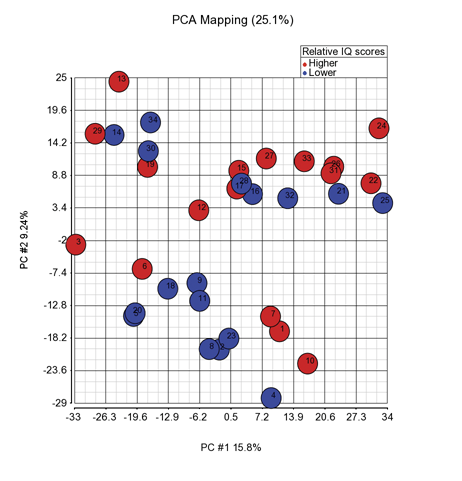

Supplement: Figure S3 — PCA results for the expression profiles of 17 twin pairs. Principle components ranked from the highest variance are named accordingly as PC 1st and PC 2nd. The PCA projection maps these two components data to 2 dimensions for visualization. In the scatter plots, each point represents a sample. The color of the symbol represents the relative IQ scores with red as higher and blue as lower. The number on each symbol indicates the twin pair ID. In contrast to the similarity between co-twins from each pair, no apparent gathering pattern could be recognized by the relative IQ scores. (TIF) [file pone.0047081.s008.tif]

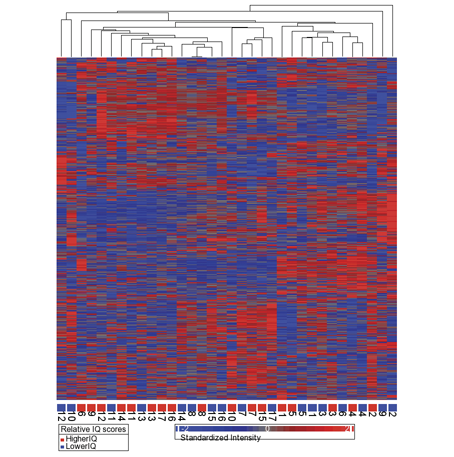

Supplement: Figure S4 — Clustering analysis for the expression profiles of 17 twin pairs. Clustering analysis for the list of 644 genes manifesting more than a 1.1-fold change between the groups of twins with higher IQ scores and the groups of their co-twins under ANOVA analysis was performed. The number below each symbol indicates the twin pair ID. The color red and blue of the symbol indicate respectively the higher IQ and the lower IQ twin of each twin pair. The scale represents fold changes, according to the color map at the bottom of the figure. No apparent clustering could be recognized. (TIF) [file pone.0047081.s009.tif]
