# Supplementary material for: Genome-Wide DNA Methylation and Gene Expression Analyses of Monozygotic Twins Discordant for Intelligence Levels
Source: PLoS One. 2012 Oct 17;7(10):e47081. doi: 10.1371/journal.pone.0047081 (PMC3474830; doi:10.1371/journal.pone.0047081)
Supplement: Table S1 — Summary of candidate loci with methylation changes identified by promoter DNA methylation patterns and their bisulfite sequencing result. (DOC) [file pone.0047081.s010.doc]

**Table S1**

**Summary of candidate loci with methylation changes identified by promoter DNA methylation patterns and their bisulfite sequencing results.**

|  |  |  |  |  |  | BS^1^: CpG methylation | |  |
| --- | --- | --- | --- | --- | --- | --- | --- | --- |
| Twin Pair | Locus | Chromosome | Strand | Fragments to BS^2^ | No.CpGs | Twin A (%) | Twin B (%) | *p** |
| 2 | *PDE11A* | 2q31.2 | - | 178681449-17868223 | 7 | 91.35 | 92.86 | 0.520145 |
|  | *TOPORS* | 9p21 | - | 32542822-32543446 | 18 | 70.93 | 71.3 | 0.893188 |
| 3 | *SLC41A2* | 12q23.3 | - | 103846690-103847314 | 2 | 100 | 100 | 1 |
|  | *GRHL1* | 2p25.1 | + | 10018030-10018654 | 6 | 88.02 | 91.67 | 0.236985 |
|  | *C3orf19* | 3p25.1 | + | 14667515-14668207 | 32 | 20.83 | 20 | 0.650596 |
|  | *B3GNT5* | 3q28 | + | 184452958-184453582 | 15 | 66.89 | 68.89 | 0.520528 |
| 4 | *PNPT1* | 2p15 | - | 55774588-55775619 | 28 | 66.55 | 63.21 | 0.152398 |
| 5 | *HECW1* | 7p13 | + | 43117880-43118577 | 15 | 22.67 | 24 | 0.636309 |
|  | *GTF3C3* | 2q33.1 | - | 197372946-197373606 | 30 | 93.67 | 92.44 | 0.307766 |
| 6 | *MOBKL3* | 2q33.1 | + | 198087792-198088416 | 6 | 12.78 | 15.56 | 0.524146 |
|  | *N6AMT1* | 21q21.3 | - | 29179862-29180560 | 15 | 98.44 | 99.11 | 0.362763 |
| 8 | *OR4D10* | 11q12.1 | + | 59000591-59001215 | 3 | 93.75 | 83.33 | 0.0234658† |
| 9 | *ARHGAP18* | 6q22.33 | - | 130073123-130073935 | 19 | 42.56 | 28.27 | 0.000000051† |
|  | *ZNF212* | 7q36.1 | + | 148566701-148567502 | 20 | 22.83 | 22 | 0.729263 |
|  | *MYEF2* | 15q21.1 | - | 46258086-46258710 | 7 | 86.67 | 80.48 | 0.0869067 |
| 10 | *KRTAP19-6* | 21q22.1 | - | 30834796-30835420 | 9 | 92.36 | 89.24 | 0.194503 |
| 12 | *KCNH1* | 1q32.2 | - | 209374441-209375065 | 12 | 71.1 | 64.44 | 0.0556305 |
|  | *PRPF4B* | 6p25.2 | + | 3965616-3966446 | 33 | 50.2 | 48.41 | 0.411187 |
|  | *TRIM60* | 4q32.3 | + | 166171777-166172432 | 19 | 97.1 | 98.3 | 0.17291 |
|  | *CSMD3* | 8q23.3 | - | 114458752-114459448 | 4 | 88.71 | 93.55 | 0.180237 |
| 13 | *GP2* | 16p12 | - | 20246552-20247176 | 4 | 90.48 | 88.69 | 0.592122 |
|  | *NOMO1* | 16p13.11 | + | 14834247-14834871 | 16 | 66.33 | 64.72 | 0.593057 |
| 14 | *TSN* | 2q21.1 | + | 122228689-122229401 | 14 | 26.67 | 28.81 | 0.487932 |
|  | *HIST1H2AA* | 6p22.2 | - | 25833379-25834039 | 13 | 95.28 | 96.67 | 0.359446 |
| 15 | *RBM28* | 7q32.1 | - | 127771314-121112154 | 17 | 69.3 | 68.2 | 0.674732 |
|  | *MRP63* | 13q12.11 | + | 20647582-20648206 | 14 | 59.68 | 58.76 | 0.782339 |
| 17 | *PCDHA4* | 5q31 | + | 140165949-140166573 | 1 | 25.81 | 32.26 | 0.7802 |

At least 30 clones per locus were sequenced.

^1^BS: bisulfite sequencing

^2^Genome coordinates relative to the NCBI Build 36 genome assembly

* p for each locus was calculated by chi-square test, except that for PCDHA4 Fisher's exact test was applied considering its small observed number.

† significant p after Bonferroni correrction (i.e., 0.05 divided by the number of loci sequenced for each pair, which is 0.05 for Twin Pair ID 8 and 0.0167 for Twin Pair ID 9).
