# Supplementary material for: Genome-Wide DNA Methylation and Gene Expression Analyses of Monozygotic Twins Discordant for Intelligence Levels
Source: PLoS One. 2012 Oct 17;7(10):e47081. doi: 10.1371/journal.pone.0047081 (PMC3474830; doi:10.1371/journal.pone.0047081)
Supplement: Table S2 — Up-regulated gene sets in the group of twins identified by GSEA (FDR q -value<0.25). (DOC) [file pone.0047081.s011.doc]

**Table S2**

**Up-regulated gene sets in the group of twins identified by GSEA (FDR q-value<0.25).**

| **Up-regulated in** | **Gene sets database** | **Gene set** | **FDR *q*-value** | **Genes with core enrichment** |
| --- | --- | --- | --- | --- |
| Higher IQ Twins | BioCarta | n/a | n/a | n/a |
|  | KEGG | One carbon pool by folate | 0 | *SHMT1, MTHFD2, ALDH1L1, TYMS, SHMT2, GART, MTHFD1, MTHFD1L, ATIC* |
|  | Reactome | n/a | n/a | n/a |
|  | Gene Ontology | Organellar ribosome | 0.035 | *MRPS35, MRPL23, MRPL52, MRPS22, MRPL55, MRPL41, MRPL12, MRPS15* |
|  |  | Mitochondrial ribosome | 0.0378 | *MRPS35, MRPL23, MRPL52, MRPS22, MRPL55, MRPL41, MRPL12, MRPS15* |
|  |  | Ribosomal subunit | 0.041 | *MRPS35, MRPL23, MRPL52, MRPS22, MRPL55, MRPL41, MRPL12, MRPS15* |
|  |  | ATP-dependent DNA helicase activity | 0.164 | *XRCC5, XRCC6, DHX9, PIF1, G3BP1, RUVBL2, CHD4* |
| Lower IQ Twins | BioCarta | n/a | n/a | n/a |
|  | KEGG | Alpha linolenic acid metabolism | 0.309 | *PLA2G2A, PLA2G2F, PLA2G12A, PLA2G4E, PLA2G4A, PLA2G1B, PLA2G12B* |
|  | Reactome | CREB phosphorylation through the activation of CAMKII | 0.228 | *GRIN2C, CAMK2G, CAMK2A, GRIN2A, CREB1, CAMK2B* |
|  | Gene Ontology | n/a | n/a | n/a |

ES, enrichment score; NES, normalized ES; n/a indicates no gene set met the cutoff FDR q-value<0.25
