# Supplementary material for: Genome-Wide DNA Methylation and Gene Expression Analyses of Monozygotic Twins Discordant for Intelligence Levels
Source: PLoS One. 2012 Oct 17;7(10):e47081. doi: 10.1371/journal.pone.0047081 (PMC3474830; doi:10.1371/journal.pone.0047081)
Supplement: Table S3 — Methylation profiling by bisulfite sequencing for IGF1 (Chr2:101335584–131398508) in Twin Pair ID 7. (DOC) [file pone.0047081.s012.doc]

**Table S3**

**Methylation profiling by bisulfite sequencing for IGF1 (Chr2:101335584-131398508^1^) in Twin Pair ID 1, 2, 7, and 17**

|  |  | Bisulfite Sequencing CpG methylation | | | |
| --- | --- | --- | --- | --- | --- |
|  | Expression level | Promoter P1 (101398180-101400138) ^1^: 10 CpGs | | Promoter P2 (101396468-101397238) ^1^: 5 CpGs | |
| Twin Pair ID | Twin A: Twin B | Twin A: Twin B | p*^2^* | Twin A: Twin B | p*^2^* |
| 1 | 1.148945: 1 | 51.33%: 52.67% | 0.743774 | 16%: 13.64% | 0.318116 |
| 2 | 2.166866: 1 | 65%: 56.33% | 0.0297869 | 6.38%: 13.64% | 0.0694191 |
| 7 | 2.328047: 1 | 56.67% : 62.67% | 0.134145 | 18%: 13.33% | 0.266199 |
| 17 | 1.212202: 1 | 54.67%: 54.67% | 1 | 19.05%: 10% | 0.122327 |

30 colonies per locus were directly sequenced

^1^fragments sequenced; genome coordinates are relative to the NCBI Build 36 genome assembly

^2^p for each locus calculated by chi-square test; after Bonferroni correction for the number of loci sequenced for each pair, p < 0.025 is considered significant
